# Schistosomiasis vaccine SchistoShield® induces functional immune memory responses in US and African populations

**DOI:** 10.1038/s41541-026-01501-0

**Published:** 2026-06-02

**Authors:** Mumtaz Y. Balkhi, Aravindan Kalyanasundaram, Aryandra Arya, Desalegn W. Kifle, Mallory Mckinnon, Dinesh Krishnappa, Harshvardhan Shrivastava, Mohamadou Siribie, Asma Aziz, Hyonjin Jeon, Tanyada Yongchaitrakul, Birkneh T. Tadesse, Njariharinjakamampionona Rakotozandrindrainy, Ravomialisoa Razafimanantsoa, Henry Bere Noellie, Nebié Issa Ouedraogo, Amidou Diarra, Sean A. Gray, Rhea N. Coler, Jinhee Lee, Raphaël Rakotozandrindrainy, Sodiomon B. Sirima, Lisa A. Jackson, Florian Marks, Darrick Carter, Afzal A. Siddiqui

**Affiliations:** 1 Department of Immunology and Molecular Microbiology, Texas Tech University Health Sciences Center, Lubbock, TX USA; 2 Center for Tropical Medicine and Infectious Diseases, Texas Tech University Health Sciences Center, Lubbock, TX USA; 3 International Vaccine Institute, SNU Research Park, 1 Gwanak-ro, Gwanak-gu, Seoul, Republic of Korea; 4 Madagascar Institute for Vaccine Research, University of Antananarivo, Antananarivo, Madagascar; 5 Cambridge Institute of Therapeutic Immunology and Infectious Disease, University of Cambridge School of Clinical Medicine, Cambridge, UK; 6 Heidelberg Institute of Global Health, University of Heidelberg, Heidelberg, Germany; 7 Groupe de Recherche Action en Santé, Ouagadougou, Burkina Faso; 8 PAI Life Sciences Inc, Seattle, WA USA; 9 Center for Global Infectious Disease Research, Seattle Children’s Research Institute, Seattle Children’s Hospital, Seattle, WA USA; 10 Quratis Inc., Osong Bioplant, 625-19, Yeonje-ri, Osong-eup, Heungdeok-gu, Cheongju-si, Chungcheongbuk-do Republic of Korea; 11 Kaiser Permanente Washington Health Research Institute, Seattle, WA USA; 12 The Hong Kong Jockey Club Global Health Institute, Hong Kong Special Administrative Region, Hong Kong, China

**Keywords:** Diseases, Immunology, Microbiology

## Abstract

Helminth parasites of the genus *Schistosoma* cause 290,000 deaths annually, mostly in tropical and subtropical regions. An estimated 250 million people are currently chronically infected with *Schistosoma* parasites, imposing a risk of new and recurrent infections in an additional 800 million people. SchistoShield^*®*^ (Sm-p80 + GLA-SE) is a leading vaccine candidate for schistosomiasis that has successfully completed Phase 1 (USA) and Phase 1b (Africa) safety and immunogenicity clinical trials. Using Peripheral Blood Mononuclear Cells (PBMCs) obtained from intercontinental Phase 1 and Phase 1b trial participants, adaptive immune effector and memory responses to SchistoShield^*®*^ were investigated. Functional recall responses were measured in vitro using Sm-p80 vaccine antigen. Results clearly demonstrate that the vaccine induced pronounced effector and memory T-cell responses. Upon recall with Sm-p80 antigen, cytokines including IFN-γ, TNF-α, IL-17A, IL-9, and granzyme B were produced, indicating the generation of functionally heterogeneous CD4 T-helper and cytotoxic lymphocyte responses. Consistent with T-helper responses that promote humoral immunity, Sm-p80 antigen-specific antibody-secreting plasmablasts were detected in vaccinated volunteers who were tracked longitudinally. Taken together, the SchistoShield® vaccine induced robust cell-mediated effector and memory responses, hallmarks of a potentially efficacious vaccine against schistosome/helminth parasites.

## Introduction

SchistoShield^®^ is a potent schistosomiasis vaccine, the core antigen of which is the large subunit of *Schistosoma mansoni* calcium-activated neutral protease (calpain), referred to as Sm-p80^1–3^. SchistoShield^®^ has been extensively tested in numerous animal models and has consistently demonstrated effective protection for all of the intra-mammalian schistosome life cycle stages^4–12^. Phase 1 and Phase 1b safety and dose-escalating clinical trials with SchistoShield^®^ have been successfully concluded in the United States^13^ and in two African countries (Madagascar and Burkina Faso). Clinical Study Reports for these trials have confirmed that the vaccine was well-tolerated without any serious safety concerns in all of the cohorts. SchistoShield^®^ elicited robust antigen-specific antibody titers in human trials^13^. Vaccine-specific antibodies generated in humans were able to effectively kill the larval stages of the parasite (schistosomula) in ex vivo assays^14^. In addition, sera from volunteers vaccinated with SchistoShield^®^ (USA and Africa) were able to successfully neutralize the enzymatic activity associated with schistosome calpain in a quantitative assay^15^. Sm-p80-specific antibody-mediated inhibition of the enzymatically active component of Sm-p80 underscores the potential of this readout to serve as a correlate of protection for SchistoShield^®^^1,15^.

To further advance the development of the SchistoShield^®^ vaccine, a cross-continental investigation was conducted to decipher the immunological memory generated in response to the vaccination in both disease-naïve and schistosome-endemic countries. Long-lived plasma cells provide serological memory and secrete high-affinity antibodies during reinfections^16–18^. Correspondingly, T-cell help is vital for vaccine-mediated humoral immunity and antimicrobial effector responses^19,20^. Antigen-reactive CD4^+^ and CD8^+^ T-cells undergo clonal expansion followed by contraction due to apoptosis, leaving behind a small subset of memory T-cells. These memory T-cells differentiate into effector memory (T_EM_) and central memory (T_CM_) subsets, distinguished by their expression of the CCR7 homing receptor^21–23^. Our understanding of T-cell-mediated immune responses to *S. mansoni* infections in endemic populations is limited. However, investigations have revealed a predominance of mixed T_H_1/T_H_2 responses in the acute phase of schistosome infection, which is followed by a transient phase, dominated by a T_H_2 response, and as the infection progresses to a chronic disease phase, a stable T-regulatory response dominates^24–26^. Chronic phase-PBMCs demonstrate hyporesponsiveness to restimulation in vitro as well as suppression of IFN-γ production^24,27^. CD4^+^ T-cell hyporesponsiveness, manifested as failure to produce effector cytokines, is seen after repeated exposure to *S. mansoni* larvae^28^.

Data obtained in the present study clearly demonstrate that the SchistoShield^®^ vaccine induces T-effector and functional immune memory responses across trials in the US and Africa. Functional memory is demonstrated by the release of T-helper cytokines upon antigen recall. We analyzed, compared and quantified the magnitude of cytokine response with and without antigen recall, assessing the quality and durability of the functional T-cell responses. Along with the T-helper response, the generation of SchistoShield^®^ vaccine-specific antibody secreting plasmablast cell populations were assessed.

## Results

### Induction of recall responses in CD4^+^ and CD8^+^ T-cells in vitro

**Fig. 1:**
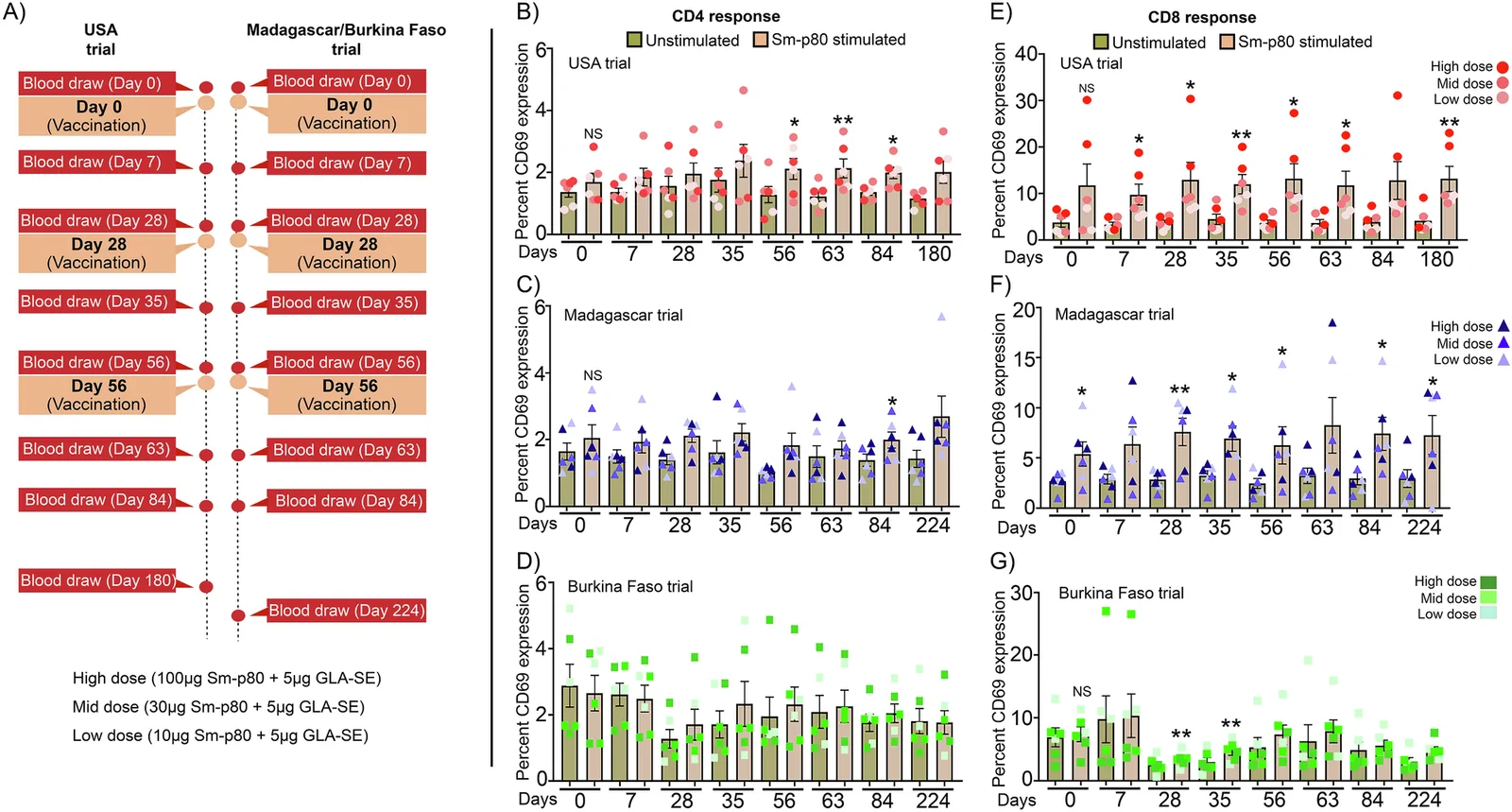
Trial design and longitudinal tracking of T-cell activation in an in vitro antigen recall. **A** Schematic illustrates the blood sample collection and SchistoShield^®^ vaccine administration time points in human participants across the US and Africa trials. In total eight blood samples were collected and three vaccine doses administrated. The three different vaccine dose concentrations are shown in the bottom panel. The volunteers in US and African trials were longitudinally tracked for 26-weeks and 32-weeks respectively. Flow cytometry of CD4^+^ (**B**–**D**) and CD8^+^ (**E**–**G**) T cells showing percentage expression of CD69 upon antigen recall in vitro. Each PBMC sample was divided into two sets, one set of cells was stimulated with Sm-p80 (12 µg) whereas second set was left untreated. The cell density of 2×10^6^ /mL was maintained equally in each set. Cells were maintained in culture with and without antigen for 72-hours. The color intensity and shapes correspond to low, mid, and high dose vaccinated volunteers. Graphs denote mean ± SEM. *p < 0.05, **p < 0.01 (*p*aired two-tailed t-test).

To delineate vaccine-induced effector and memory responses, experiments were performed to examine T-cell recall responses to the Sm-p80 antigen in vitro using PBMCs from participants in the three trials (USA, Madagascar and Burkina Faso). These three trials were carried out utilizing an identical dosing and inoculation regimen (Fig. 1A). Compared to the pre-vaccine baseline (day 0), post-vaccinated samples revealed upregulation of activation marker CD69 on both CD4^+^ and CD8^+^ T-cells after stimulation with the Sm-p80 antigen (Fig. 1). A 40% increase in CD69 expression was observed on CD4^+^ T-cells across US and Madagascar trial participants (Fig. 1B, C). Similarly, CD8^+^ T-cells exhibited 20% and 40% CD69 upregulation in samples from these trials, respectively (Fig. 1E, F). On day 0 and day 8, CD4 + T-cells from Burkina Faso trial participants had elevated baseline expression, likely due to the endemic nature of the study population and potentially prior parasite exposures. However, at later time points (excluding days 0 and 8), there was a clearer differential T-cell response to the Sm-p80 antigen in samples from Burkina Faso participants. CD69 expression increased to 20% for CD4^+^ T-cells (Fig. 1D) and 33% for CD8^+^ T-cells (Fig. 1G).

### Cytokine responses to Sm-p80 antigen recall in vitro

**Fig. 2:**
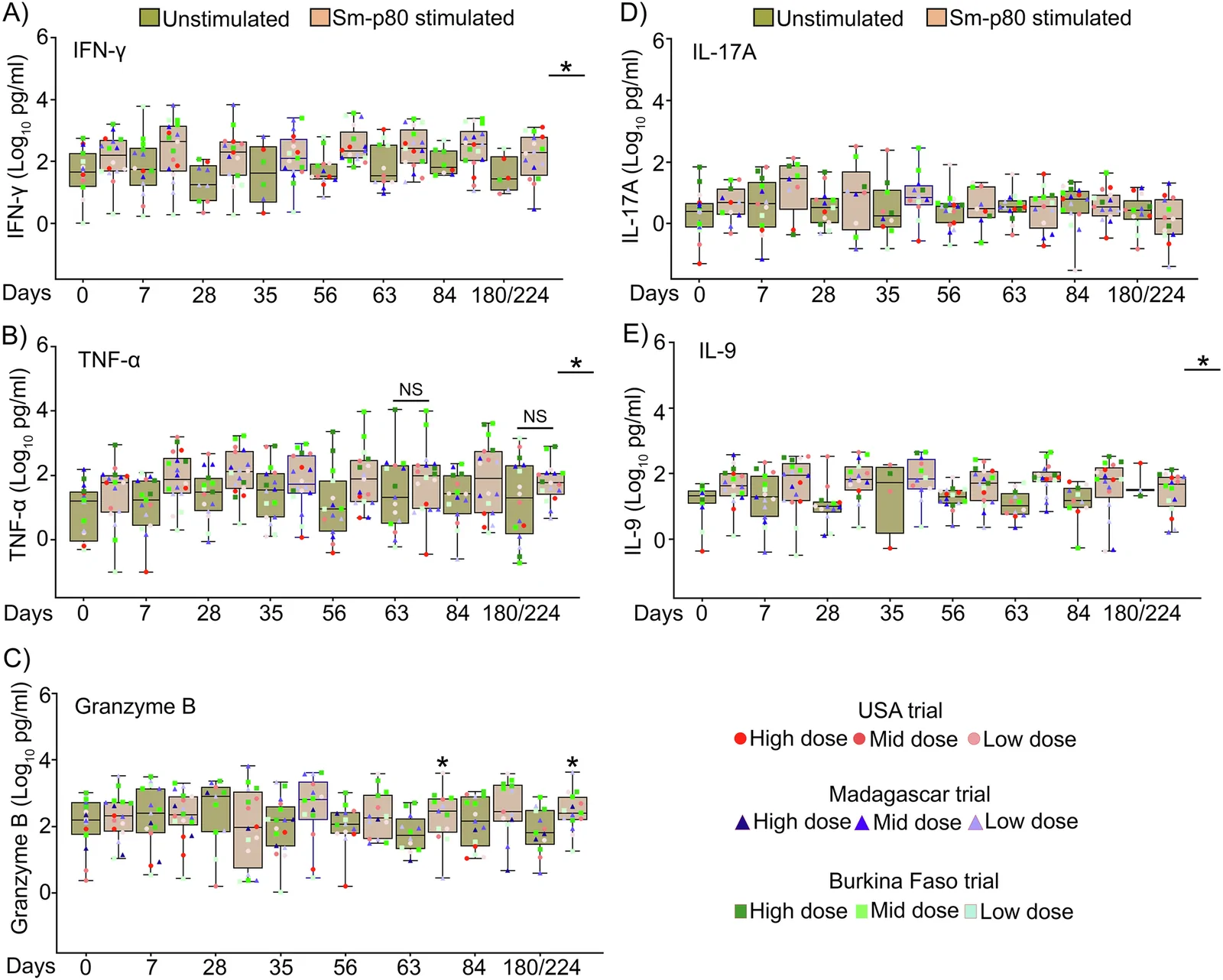
Cytokines detected in response to Sm-p80 antigen recall in vitro. Cytokine response to Sm-p80 antigen recall was measured using CodePlex/IsoPlexis system. The PBMCs collected from the volunteers across all eight time points were cultured and stimulated as described. The supernatants, both stimulated and corresponding unstimulated cultures, were analyzed. Shown is the response data for IFN-γ, TNF-α, Granzyme B, IL-17A and IL-9 production (**A**–**E**). The values on y-axis were log_10_ transformed. In total all 18-volunteers, six each from US, Madagascar and Burkina Faso trial (represented by different shapes) were analyzed together. The missing shape denote the volunteers in which cytokine expression was not detected. Graphs denote mean ± SEM. Wilcoxon matched-pairs signed rank test was used to determine statistical significance in data comparing untreated and Smp80 stimulated PBMCs. The underlined * represent the *p*=values that are statistically significant across all times points. NS not significant.

To assess functional responses to the Sm-p80 antigen in vitro, the magnitude of cytokine production in culture supernatants was quantified across all three trials using the high-throughput CodePlex/IsoPlexis adaptive cytokine assay. Supernatants from all eight unstimulated and corresponding Sm-p80-stimulated cultures (Fig. 1A) were examined simultaneously. A robust and long-lasting production of several cytokines in response to Sm-p80 antigen was detected. Levels of T_H_1 effector cytokines, IFN-γ and TNF-α, in culture supernatants increased upon Sm-p80 antigen recall and remained elevated following booster doses (Supplementary Table 1). On day 0, a baseline average of 300 pg/mL of IFN-γ was detected following Sm-p80 stimulation. In comparison, post-vaccination day 7, IFN-γ production increased to 940 pg/mL. The cytokine levels were maintained in the range of 600 pg/mL throughout before declining to 320 pg/mL in samples collected at the last time point (Fig. 2A). Similarly, levels of TNFα production were sustained for a prolonged duration. On day 0, a baseline TNFα level of 100 pg/mL was detected using the Sm-p80 antigen, and the level increased to 280 pg/mL on day 7. Following the second boost, TNFα concentration peaked at 700 and 850 pg/mL on day 63 and 84, respectively, before declining to 130 pg/mL in the samples collected at the last time point (Fig. 2B). The T_H_1 effector cytokine IL-2 was also detected, primarily in *–* PBMC culture supernatants. IL-2 levels increased relative to day 0 baseline at day 7 (one week post-primary vaccination) and day 35 (one week post-first boost), as shown in Supplementary Fig. 3. This is consistent with the kinetics of vaccine-induced T-cell effector phase response, which typically lasts one to two weeks before the contraction and resting phases. Granzyme B was also detected in culture supernatants (Fig. 2C), with levels increasing following the first and second boost and remaining elevated relative to day 0 upon antigen recall through the final time point, indicating a sustained cytotoxic lymphocyte response to vaccination. In addition to T_H_1 cytokines, SchistoShield^®^ induced T_H_17 and T_H_9 responses, as indicated by the production of IL-17A and IL-9, respectively, in the culture supernatants upon antigen recall. IL-17A showed an initial higher release followed by a slow decline post-first boost (Fig. 2D). Similarly, IL-9 levels in culture supernatants increased post-primary vaccination day 7 relative to day 0 following antigen recall (74 pg/mL to 100 pg/mL) and then remained stable at 100 pg/mL before declining in samples collected at last time point (Fig. 2E). Both IL-17A and IL-9 have demonstrated anti-helminthic activities in various in vivo models. Overall, the cytokine data are consistent with functional T-cell responses across all of the three trials.

### Vaccine-specific CD4^+^ effector memory T-cells

**Fig. 3:**
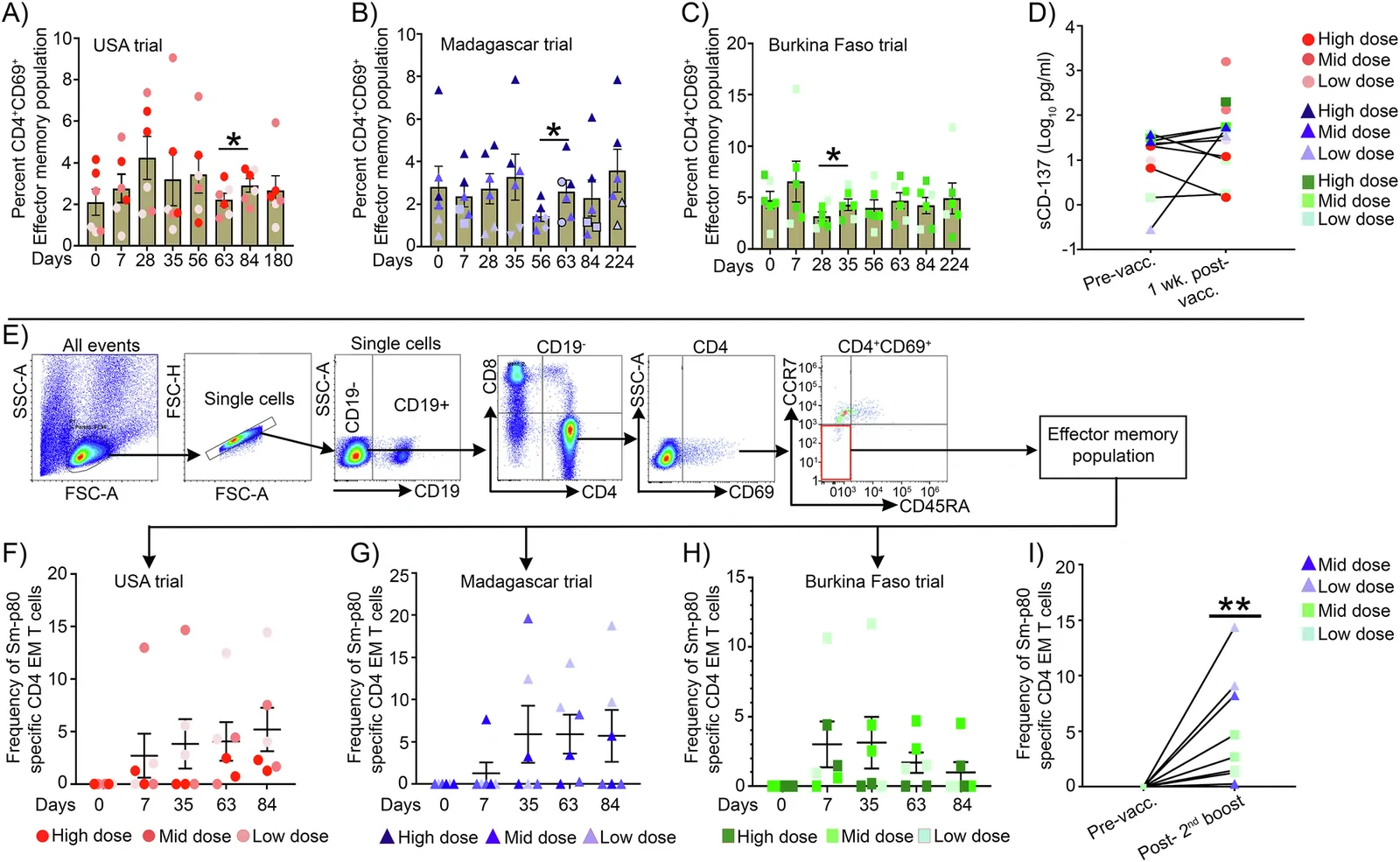
SchistoShield^®^ vaccine generates CD4^+^ effector memory cells. **A**–**C** Flow cytometry analysis showing magnitude of CD69 activation marker expression on CD4^+^ effector memory pre- and post-vaccination tracked longitudinally across all three trials. **D** sCD-137 in culture supernatants of PBMCs pre- and one week post-vaccination was detected through CodePlex/Isoplexis cytokine analysis (n = 18). **E** Show flow cytometry gating strategy adopted to examine Sm-p80 antigen specific CD4^+^ effector memory population. **F**–**H** PBMCs across three trials were cultured in vitro in presence of Sm-p80 for 72-hours, following which CD4^+^ T-cells were examined for CD4^+^ effector memory population by subtracting the Sm-p80 reactive cells in pre-vaccinated cultures. **I** CD4^+^ effector memory profile of low and mid dose vaccinated volunteers consisting of Africa trials demonstrating statistically significant expansion of memory cells following second booster dose (day 63) when compared to pre-vaccinated (day 0) samples. Both day 0 and day 63 samples were stimulated with Sm-p80. Paired Wilcoxon Signed Rank test was for applied for (**I**). All graphs denote mean ± SEM. **p < 0.01.

To examine SchistoShield^®^ vaccine-generated antigen-specific CD4+ memory T-cells, PBMCs collected from eight time points across all three trials were analyzed using flow cytometry. After excluding dead cells, doublets, CD8- and CD19-positive populations, CD4^+^ T-cells were analyzed for an effector memory phenotype using the CD45RA^-^CCR7^-^ marker. The expression of the activation marker CD69 on the total CD4^+^ effector memory population was assessed using flow cytometry. The CD4^+^ effector memory population after vaccination is known to induce CD69. In samples from the US trial, an upregulation of CD69 expression was observed on CD4^+^ effector memory T cells compared with day 0. Expression reached peak levels on days 29 and 56, followed by a decline to 30% on day 181 (Fig. 3A). CD69 expression was then assessed in the total CD4+ effector memory population among trial participants from schistosome-endemic Madagascar and Burkina Faso. In the Madagascar trial, a 17% upregulation of CD69 expression was observed after the first boost (day 35) relative to day 0, reaching to 27% by the final collection point (day 224). However, a comparison between day 56 samples and 63 (one week post-second boost) revealed a significant upregulation (*p* < 0.05) of CD69 – approximately 81% (Fig. 3B). Likewise, in the Burkina Faso trial, a 37% upregulation of CD69 was observed one week post-primary vaccination compared to the pre-vaccinated day 0 baseline. One week post-first boost (day 35), CD69 was upregulated by 36% (p < 0.05) compared to day 28 post-primary vaccination (Fig. 3C).

In addition to the CD69 activation marker, sCD-137 in culture supernatants was also examined using the CodePlex/IsoPlexis assay system. Following SchistoShield^®^ vaccination, upregulated sCD-137 expression was detected in the majority of trial participants across all three trials in paired comparisons (Fig. 3D).

To identify Sm-p80 antigen-specific CD4^+^ effector memory cells, PBMC were stimulated with Sm-p80 antigen for three days in culture and collected for flow cytometry analysis, applying the gating strategy shown in Fig. 3E. The Sm-p80-induced CD69 activation marker on the CD4^+^ (CD4^+^CD69^+^) population was gated to identify the Sm-p80 antigen-specific effector memory population. In the US trial, 40% and 50% increase in the frequency of effector memory population post-first boost (day 35) and post-second boost (day 63), respectively, was observed compared to one week post-primary vaccination (day 7). The frequency of memory cells peaked on day 84 in all participants examined (Fig. 3F), indicating a progressive expansion of effector memory cells with booster doses. The antigen-specific CD4^+^ effector memory population was assessed in PBMCs from participants in the Madagascar and Burkina Faso trials; the percent increase in effector memory markers was greater post-second boost (day 63) than at primary vaccination (day 7; Fig. 3G, H). When volunteers from endemic regions (Madagascar and Burkina Faso) in the low- and mid-dose cohorts were examined together, the frequency of Sm-p80-specific CD4^+^ effector memory population increased significantly following the second boost (day 63) compared to pre-vaccination baseline (day 0; paired comparison; Fig. 3I).

### Expansion of CD8^+^ TEMRA and effector memory population

**Fig. 4:**
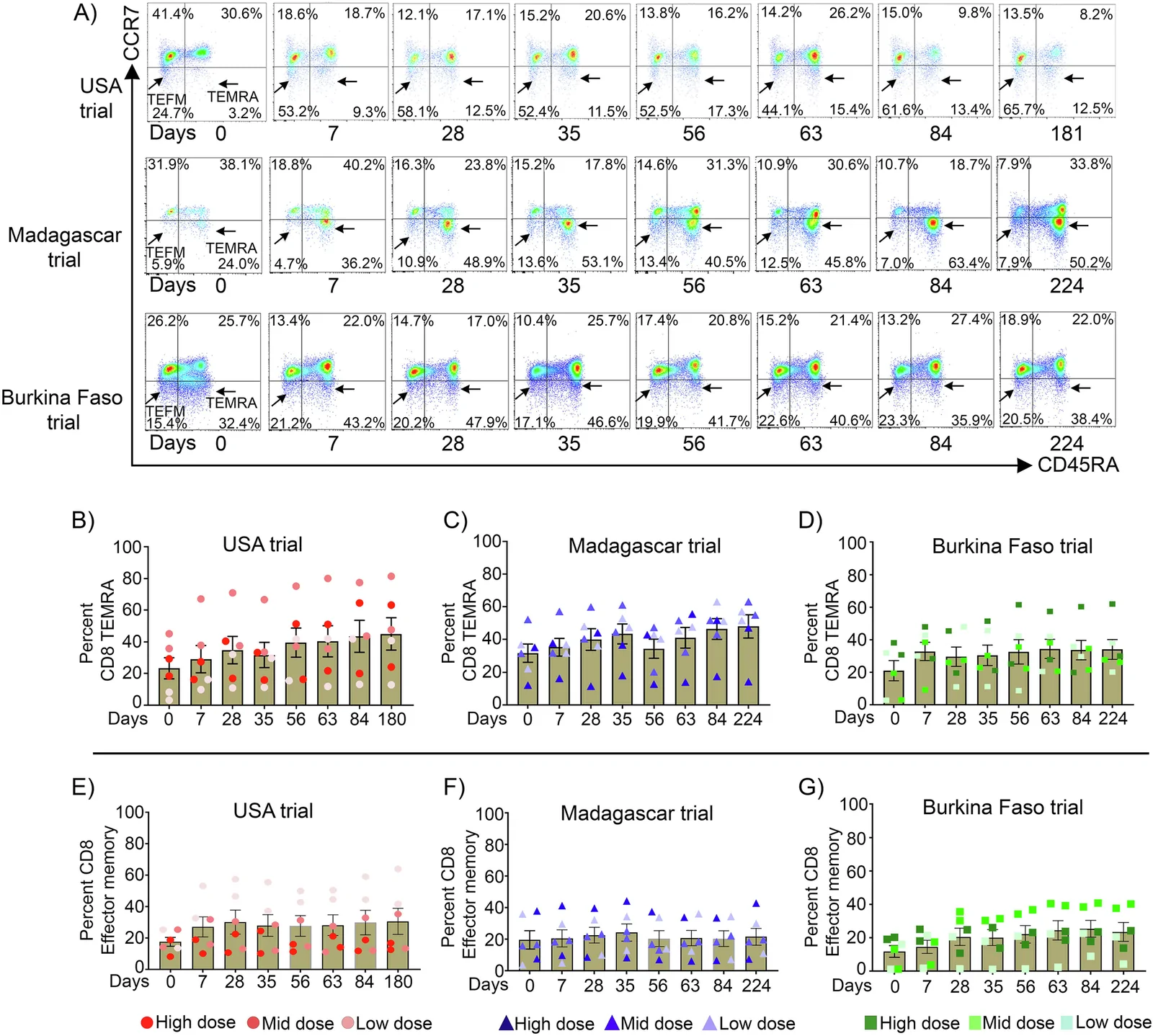
SchistoShield^®^ vaccine induces expansion of CD8^+^ TEMRA and effector memory population. **A** A representative longitudinally tracked flow cytometry profile of volunteers injected with low dose SchistoShield^®^ showing CD8^+^ memory compartments across all three-trial participants. Arrows point to CD8^+^_(TEMRA)_ and CD8^+^_(EM)_ population showing an incremental improvement in the magnitude of these memory subsets. **B**–**D** Show percentage of CD8^+^ TEMRA population obtained through flow cytometry combining data from all participants of US (n = 6), Madagascar (n = 6) and Burkina Faso (n = 6) trials. A one-way ANOVA for US trial= **p < 0.01; Madagascar trial = *p < 0.05. For Burkina Faso (*p* < 0.08). **E**–**G** percentage of CD8^+^ effector memory population. One way ANOVA for all three trials was not statistically significant. Graphs denote mean ± SEM.

To decipher the role of CD8^+^ TEMRA (terminally differentiated effector memory re-expressing CD45RA) and conventional effector memory cells, after a thaw and rest, PBMC were cultured for 72 hours. The cells were analyzed using flow cytometry, and after excluding CD4^+^, CD19^+^, and doublets, the cells were gated on the CD8^+^ population. CD8^+^ T-cells were analyzed for a memory phenotype using CD45RA and CCR7 markers. The CD8^+^ TEMRA, defined as CD45RA^+^CCR7^-^, were examined first (Fig. 4A and Supplementary Fig. 4). In the US and Madagascar trials participant samples, a significant expansion of the CD8^+^ TEMRA population was observed (one-way ANOVA *p* < 0.01). The longitudinal improvement in the population expansion compared to pre-vaccination (day 0) reached to a maximum of 1.93-fold (US trial) and 1.5-fold (Madagascar trial) on the last day of sample collection (Fig. 4B-C). A similar expansion of the CD8^+^ TEMRA population (1.62-fold) was also observed for the Burkina Faso trial participants, although the improvement was not statistically significant (one-way ANOVA *p* = 0.08) (Fig. 4D). Correspondingly, the expansion of the CD8^+^ effector memory population (CD45RA^-^CCR7^-^) was also examined. Similar to TEMRA, a longitudinal expansion of CD8^+^ effector memory population was observed compared to day 0 across all of the three trials (Fig. 4A, E-G and Supplementary Fig. 4). For the US trial, a 1.74-fold expansion was detected post-second boost (Fig. 4E). For the participants of the Madagascar trial, unlike TEMRA, expansion of CD8^+^ effector memory population was modest reaching a maximum of 1.24-fold post-first boost followed by a decline (Fig. 4F). The CD8^+^ effector memory population in the participant samples from the Burkina Faso trial showed a consistent expansion of 2-fold post- second boost, which was maintained until the last time point of the PBMC collection (Fig. 4G).

**Fig. 5:**
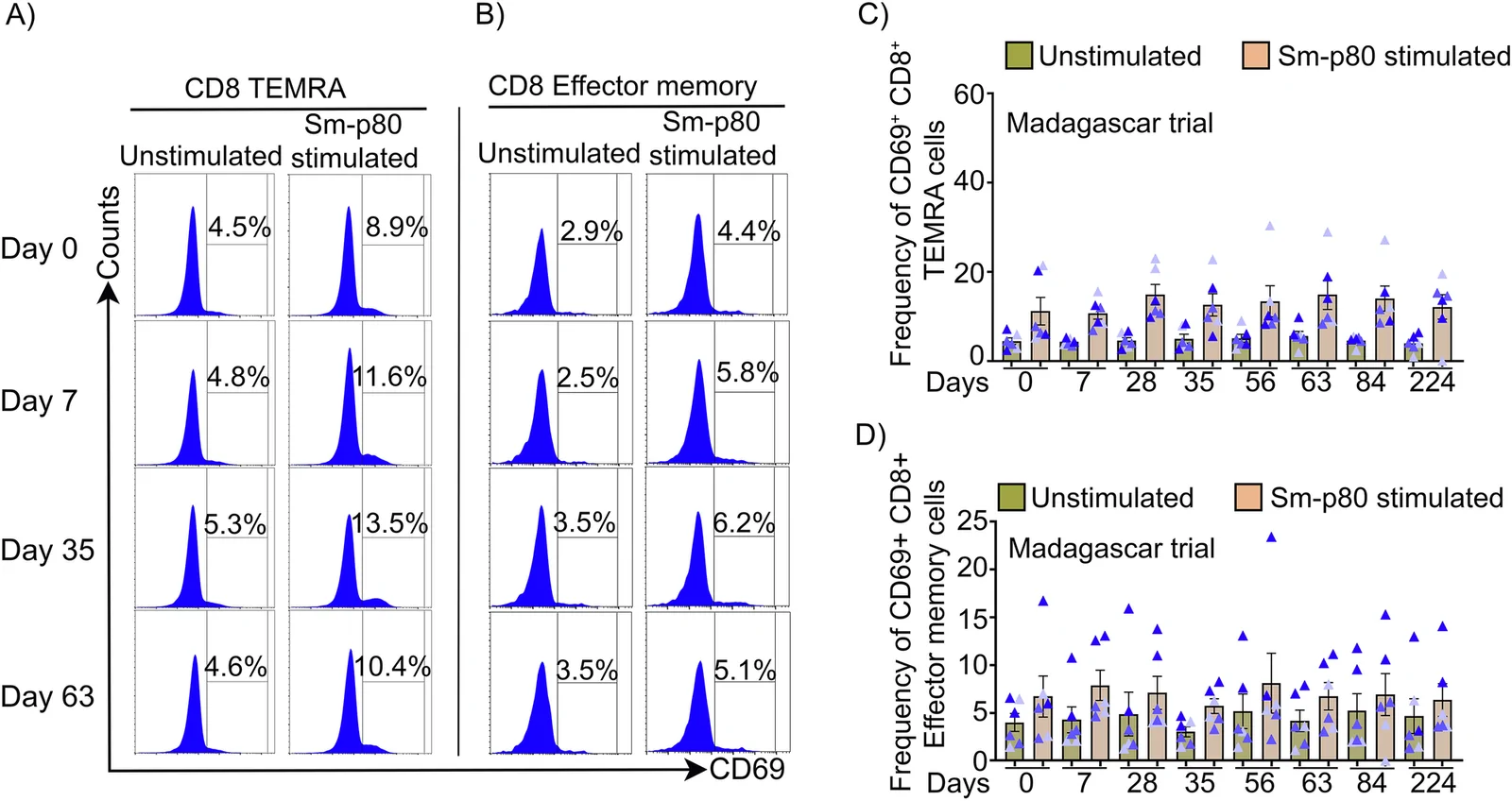
Sm-p80 induces activation of CD8^+^ TEMRA and effector memory population in the in vitro cultures. PBMCs belonging to Madagascar trial were cultured in vitro either absence or presence of Sm-p80 and followed longitudinally for CD69 expression on CD8^+^ TEMRA and effector memory subtypes using flow cytometry. **A**, **B** Representative histograms showing a consistent upregulation of CD69 activation marker with Sm-p80. **C**, **D** Shows the changes occurring longitudinally in the frequency of CD8^+^CD69^+^ TEMRA and effector memory population with Sm-p80.

Recognizing that a *Schistosoma-*endemic population may harbor pre-existing antigen-specific CD8^+^ memory precursors due to prior parasite exposure, experiments were designed to assess SchistoShield^®^-mediated expansion of the CD8^+^ memory population upon Sm-p80 antigen recall in vitro. PBMC from participants in the Madagascar trial were cultured in vitro. PBMC were split into two sets: one was stimulated with Sm-p80, and the other was left unstimulated. After 3 days in culture, cells were examined by flow cytometry for Sm-p80 antigen-induced CD8^+^ TEMRA, effector memory activation, and expansion. A consistent activation of both CD8^+^ TEMRA and the conventional effector memory population was observed upon Sm-p80 antigen stimulation, as evidenced by of CD69 upregulation and incremental increase in expression in post-vaccination samples (Fig. 5A, B). Furthermore, a longitudinal expansion of 1.12-fold post-first boost to high 1.32-fold post-second boost of CD8^+^ TEMRA (CD8^+^CD69^+^CD45RA^+^CCR7^-^) was observed with Sm-p80 antigen stimulation (Fig. 5C). The Sm-p80 antigen-mediated expansion of the CD8^+^ effector memory population was also examined. Like CD8^+^ TEMRA, expansion of the conventional CD8^+^ effector memory population (CD8^+^CD69^+^CD45RA^-^CCR7^-^) occurred following Sm-p80 antigenic stimulation. However, expansion reached a maximum of 1.29-fold post-second boost (Fig. 5D), which is lower than the CD8^+^ TEMRA. These data demonstrated that SchistoShield^®^ expands the *Schistosoma* antigen-reactive CD8^+^ memory compartment. However, it remains unclear whether CD8 memory expansion was driven by *Schistosoma* antigen-specific CD8 memory cells or by a paracrine bystander response. This aspect will be examined in the future during our planned phase 2 clinical studies.

### SchistoShield^®^ vaccine-specific antibody-secreting plasmablasts

**Fig. 6:**
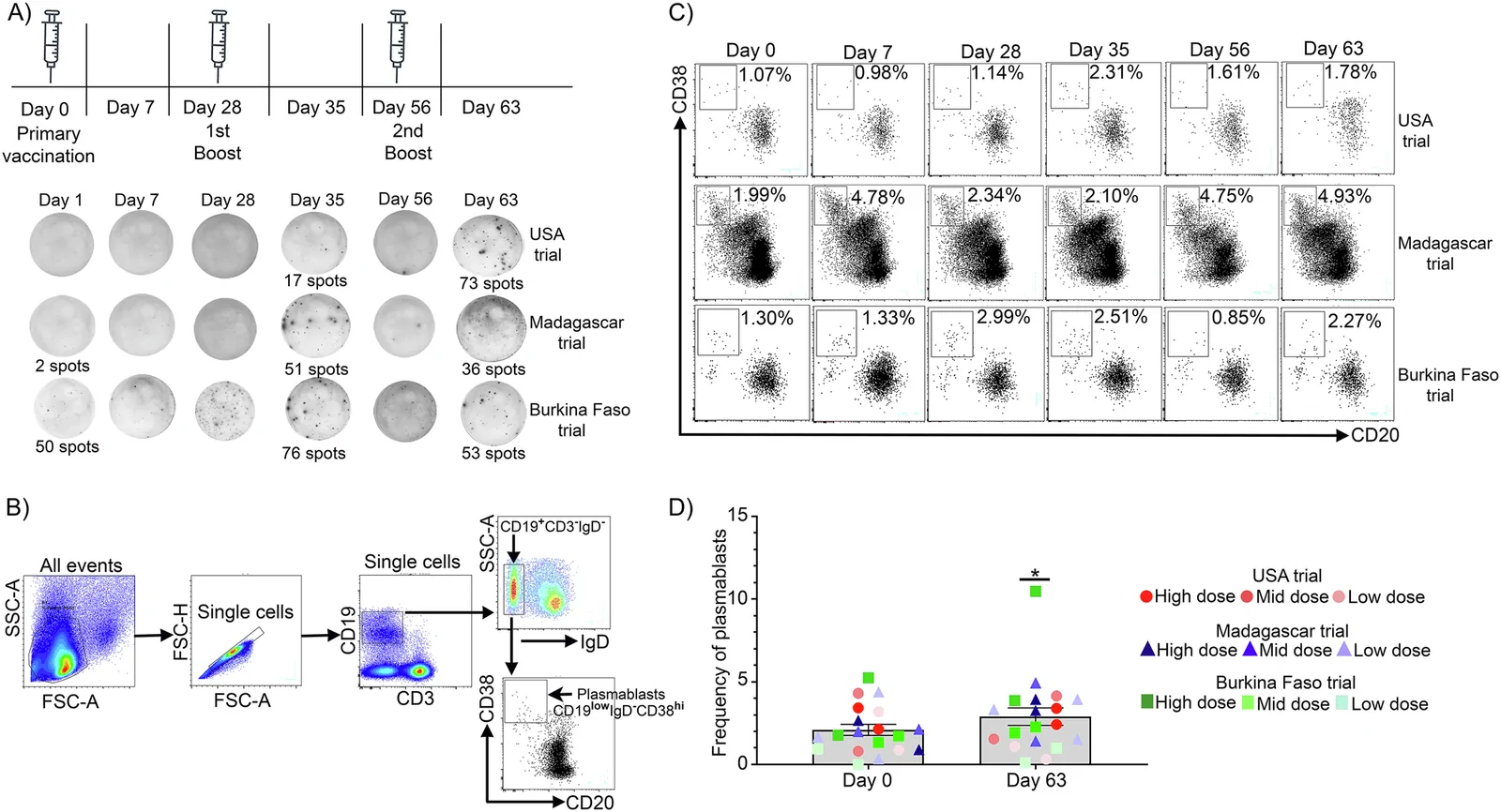
SchistoShield^®^ vaccine generates antibody secreting cells/plasmablasts. **A** ELISpot assay performed on PBMCs derived from participants across all three trials (n = 18). The ELISpot images are presented from pre-vaccinated day 0, one week (day 7), four weeks post-primary vaccination (day 28), one week post-first boost, one week pre- and post-second boost. **B** Gating strategy adopted to identify plasmablasts. **C** Representative flow cytometry profile showing CD38^++^CD19^low^CD20^-^ plasmablasts from mid-dose vaccinated volunteers. **D** Frequency of plasmablasts (CD38^++^CD19^low^CD20^-^) at pre-vaccination day 0 and following the second booster dose (day 63). Graph denote the mean ± SEM calculated from all the three trial participants. *p < 0.05.

ELISpot assays were performed to detect Sm-p80 antigen-specific antibody-secreting cells (ASC) or plasmablasts in vaccinated volunteers. PBMCs from all three trials were cultured simultaneously without any cytokine supplementation. ELISpot plates were coated with Sm-p80 antigen and incubated with PBMC from trial participants. The ELISpot data demonstrated the presence of antigen-specific antibody-secreting cells as follows: In the US trial, day 0 pre-vaccinated PBMCs showed no reactivity to the Sm-p80 antigen, consistent with the non-endemic, antigen-naïve population. However, after the first boost, antigen-specific reactivity was observed. The number of spots detected increased with a second boost, suggesting potential maturation of antigen-specific antibody affinity (Fig. 6A ELISpot, US trial). Similar results were obtained with endemic populations from Madagascar and Burkina Faso. However, here the day 0 pre-vaccinated volunteers - in contrast to the non-endemic US population - did show reactivity with the Sm-p80 antigen; however, the reactivity increased after booster doses (Fig. 6A ELISpot, Madagascar and Burkina Faso trials).

The presence of ASCs/plasmablasts was assessed by flow cytometry. Circulatory antibody-secreting cells were defined as CD19^low^ and CD38^hi^. The gating strategy is shown in Fig. 6B. Single cells were gated on CD19^+^ after excluding CD3^+^ T cells. The IgD^+^ population was further excluded from the CD19^+^ population. Finally, the CD19^+^IgD^-^CD3^-^ population was plotted against CD20 and CD38 markers. Plasmablasts were defined as CD19^low^CD20^-^CD38^++^. The data revealed an upregulation of plasmablasts post-vaccination. The population of plasmablasts increased significantly one week post-second boost (day 63) (Fig. 6C, D).

## Discussion

In the current study, immune effector and memory response data from three safety and immunogenicity trials of the SchistoShield® vaccine are presented. Three different Sm-p80 antigen vaccine doses, low, mid, and high, were administered. We have systemically examined each dose across the three trials for adaptive immune effector and memory responses. An expansion of antigen-specific CD4^+^ T-cell effector memory population was noted upon antigen recall. In addition to CD4^+^ T-cells, an expansion of total CD8^+^ TEMRA and effector memory cells was observed across all three trials. The significant expansion of CD8^+^ TEMRA, as well as their antigen-recall response in vitro in parasite-endemic populations from Africa, suggested a role for this memory subtype that remains to be further elucidated^29–31^. However, it would be prudent to mention that it is possible that the CD8^+^ activation and memory response in vitro could be a bystander, non-TCR response driven by cytokines in the supernatants^32^. The possibility also exists that in the endemic population, there is a generation of *Schistosoma* antigen-specific CD8^+^ memory responses via cross-presentation^33^. However, the development of CD8^+^ memory and the mechanism of the response to Sm-p80 antigen during in vitro recall in the context of SchistoShield^®^ remains to be fully elucidated. Vaccine-induced expansion of CD8^+^ memory (T_EM_ or T_EMRA_) and their contribution to pathogen clearance, analogous to their demonstrated role in antimicrobial immunity^34^, will be studied in more detail in our planned future studies. To assess functional responses to the antigen recall, high-throughput CodePlex/IsoPlexis cytokine assays were performed across all of the three trials, including supernatants collected from all eighteen volunteers, both unstimulated and Sm-p80-stimulated. CodePlex/IsoPlexis is a chip-based assay that enables simultaneous, autonomous detection of a wide array of cytokines. The cytokine data demonstrated a heterogeneous polyfunctional response across all three trials during antigen recall (Fig. 2 and Supplementary Table 1). Production of IFN-γ and TNF-α (T_H_1 cytokines) upon antigen recall in the vaccinated samples improved significantly. The IL-2 cytokine was not detected during antigen recall. However, IL-2 was detected in ex vivo PBMC culture supernatants, consistent with the kinetics of vaccine-induced T-cell effector phase response^35,36^ (Supplementary Fig. 3). Since significant activation of T-cells mediated by Sm-p80 stimulation was observed, the possibility exists that IL-2 may have been consumed rapidly from the culture supernatants, leading to its faster depletion. Moreover, sCD-137, a member of the TNF receptor family, was detected in the PBMC culture supernatants from vaccinated samples but not during antigen recall. The detection of these three cytokines, along with sCD-137, indicates the generation of polyfunctional T-cell responses to SchistoShield^®^^37–40^. Both CD4^+^ and CD8^+^ T-cells can produce T_H_1 effector cytokines in response to antigen encounter. We plan to perform an in-depth examination to identify polyfunctional T-cells in a much larger cohort of samples from our future phase 2 clinical trial. Our lab has consistently demonstrated IFN-γ production and general T_H_1 bias over T_H_2 to SchistoShield^®^ in animal models^2,41^. In addition, IFN-γ has long been known to be vital for vaccine-mediated protection to *S. mansoni*^42,43^. Schistosome infection has been associated with increased IFN-γ production during the acute phase. However, cytokine levels drop as infection progresses to the chronic disease phase^24,44^. We have confirmed this distinct pattern in a controlled *S. mansoni* human infection model^26^. Our published results demonstrated that IFN*-γ* is upregulated at the transcriptional level early during infection, followed by a decline within weeks^45^. Correspondingly, SchistoShield^®^ induced the long-lasting production of IFN-γ - along with other T-effector cytokines - in an antigen recall, which would indicate vaccination induces a signature consistent with protection. Two other cytokines of critical importance released in response to Sm-p80 antigen recall in vitro are IL-17A and IL-9. Specific subsets of T-helper cells (Th-17 and Th-9) are the major sources of these cytokines^46,47^. IL-17A is vital for antimicrobial defense at mucosal surfaces, inducing neutrophil recruitment and macrophage-mediated pathogen killing, and its production during the memory response to antigenic challenge has been associated with protection against various diseases, such as tuberculosis and pertussis^47–49^. In addition, IL-9, a member of the common γ-chain cytokine receptor family that also includes IL-2 and IL-15, has been reported to play a role in the expulsion of nematodes from the gastrointestinal tract through its binding to mucosal mast cells, leading to mucus production and promoting muscle contraction^50,51^. IL-9 has further been shown to lead to rapid expulsion of the nematode *Trichuris muris*^52^. Detection of these cytokines after antigen recall demonstrated vaccine-induced heterogeneous T-cell responses. Overall, the human cytokine data are consistent with our previously published findings in baboons, which exhibited a T_H_1-biased cytokine response and were protected against schistosmiasis^3,11,53^. Importantly, the SchistoShield^®^ vaccine appears to reverse immune hyporesponsiveness associated with long time chronic helminthic infections, including those caused by *Schistosoma* spp^28,54,55^. The relationship between T-cell memory subtypes and resistance or susceptibility to *S. mansoni* infection in endemic areas has been examined, and, in general, a lower frequency of memory CD4^+^ and CD8^+^ T-cells has been associated with greater susceptibility to schistosome infections^56,57^. Whether the expansion of CD4^+^ and CD8^+^ T-cell memory subtypes in response to the SchistoShield^®^ vaccine offers long-term protection will be studied in our future clinical trials.

The SchistoShield^®^ vaccine has consistently induced seroconversion in the US^13^ and African trials (unpublished findings). In the current study, we explored the generation of antibody-secreting cells in vaccinated volunteers. Here, our antibody-secreting cell terminology includes plasmablasts. Our data obtained from the smaller cohort of volunteers confirmed the generation of antibody secreting cells/plasmablasts across all of the three trials with antigen binding affinity of secreted antibodies improving post-second boost, a qualitative improvement consistent with conventional booster vaccination strategies and the T-helper cytokine response induced by Sm-p80 (Fig. 6). Plasmablasts mature into long-lived plasma cells and can provide protection throughout the life of individuals by secreting affinity-matured antibodies during reinfection^58,59^. The presence of SchistoShield^®^-specific long-lived plasma cells will also be studied in planned future clinical trials.

We observed no significant impact of antigen dose on the magnitude of induction of T-effector/memory or antibody secreting plasma cells/plasmablasts, consistent with low antigen doses being sufficient for cellular immunity – especially when given with a potent adjuvant. Taken together, the data in this report demonstrate that SchistoShield^®^ induces a durable CD4^+^ T-cell memory response; primes and expands the CD8^+^ memory population; and elicits a heterogeneous, polyfunctional effector cytokine response, along with vaccine-specific antibody-secreting cells. Whether these diverse components of the immune system coordinate to mediate long-term protection against schistosomiasis will be revealed once the vaccine is deployed in endemic regions. Nevertheless, data obtained from our previously published baboon study, which exhibited an analogous immune effector phenotype, demonstrated efficient protection, including a significant reduction in female worms, egg burden, egg-induced pathology and interruption of parasite transmission^3^.

## Methods

### Human samples

All of the human samples were obtained after prior informed consent of participants and ethical approvals. The first-in-human Phase 1 trial was conducted at the Kaiser Permanente Washington Health Research Institute, Seattle, WA, USA (Registration date: 23/03/2022, ClinicalTrials.gov ID, NCT05292391) using approved protocol (DMID 18-0018). The Phase 1b trials were conducted in Madagascar (Registration date: 2023-02-28, ClinicalTrials.gov ID: NCT05762393) and Burkina Faso (Registration date: 2023-03-17) using the IVI VASA 001 protocol, which was also approved by the International Vaccine Institute (IVI) Institutional Review Board (IRB number 2022-011). Clinical trials were conducted in accordance with International Council for Harmonization/Good Clinical Practices (ICH-GCP), adhering to the ethical principles of the Declaration of Helsinki and Council for International Organizations of Medical Science (CIOMS) as well as applicable local ethical and regulatory requirements. The number of human volunteer samples used in the study is eighteen, among which six each originated from the USA, Madagascar, and Burkina Faso. The volunteer samples from Africa trials were screened for circulating anodic antigen (CAA)^60^ positivity to rule out active *Schistosoma* infection.

For all of the three trials, the primary vaccine dose was administered on day 0 followed by a first and second booster dose on day 28 and 56 respectively. Blood samples were collected on day 0 (pre-vaccination), followed by day 7 and day 28 (*i.e*., one and four weeks post-primary vaccination); day 35 and day 56 (i.e., one and four weeks post-first boost); day 63 and day 84 (*i.e*., one and four weeks post-second boost). The last blood sample (eighth) was collected on day 180 (for the US trial), around eighteen weeks post- second boost, while for the Madagascar and Burkina Faso trials, the last blood sample was collected on day 224, which is twenty-four weeks post-second boost. In total eight blood samples per volunteer were collected. Three antigen doses, low (10 µg Sm-p80 + 5 µg GLA-SE), mid (30 µg Sm-p80 + 5 µg GLA-SE), and high (100 µg Sm-p80 + 5 µg GLA-SE) were tested across all of the three trials to determine if there was a dose dependence on response (Fig. 1A).

### Sm-p80 antigen

Sm-p80 is an *E. coli*-produced, recombinant protein using the sequence derived from the *S. mansoni* calpain, Sm-p80. GMP-grade Sm-p80 was manufactured by the Center for Biocatalysis and Bioprocessing at the University of Iowa under the supervision of PAI Life Sciences (lot # 0440) and was used in the clinical trials, whereas a GMP lot of Sm-p80 (QTP-105; Quratis Inc., Republic of Korea) was used for the in vitro stimulation studies^61^.

### Antibodies for flow cytometry

The following fluorochrome conjugated antibodies were used for T-cell analysis: PerCP-Cyanine5.5 CD45RA (HI100) (Cat. # 45-0458-42, Thermo Fischer), PE-CD197 (3D12) (Cat. # 12-1979-42, Thermo Fischer), APC-eFluor-780-CD4 (RPA-T4) (Cat. # 47-0049-42, Thermo Fischer), BB515-CD8^+^ (RPA-TB) (Cat. #564526, BD Horizon), APC-CD69 (L78) (Cat. # 340560, BD). For B and plasma cell analysis, we used the following antibodies: cFluor® V420 CD3 (SK7) (Cat. # R7-20053, Cytek), BV711-CD19 (SJ25C1) (Cat. # 563038, BD Horizon), CD-20 (2H7) (Cat. # 566988, BD Horizon), BB515-CD38 (HIT2) (Cat. # 564498, BD Horizon), APC/Fire™ 810-CD27 (O323) (Cat. # 302864, Biolegend), BV510-IgM (G20-127) (Cat. # 563113, BD Horizon), PE-IgG (Cat. # 130-119-878, Miltenyi Biotec), PE/Cynanine 5-IgD (IA6-2) (Cat. # 348250, Biolegend).

### CodePlex/IsoPlexis cytokine analysis

PBMC culture supernatants, unstimulated and Sm-p80 stimulated were analyzed using adaptive cytokine panel. Stored culture supernatants were thawed before being loaded into CodePlex chips (Bruker Cellular Analysis). Following the manufacturer’s instructions, 5.5 µL of supernatant from each sample were loaded in duplicate into the CodePlex chip wells. Complete RPMI medium was used as a control for the background normalization. The CodePlex chips were loaded into the IsoSpark Duo (Bruker Cellular Analysis), which autonomously detects the secreted cytokines. The secreted cytokine levels were quantified using IsoSpeak software.

### In vitro PBMC culture and flow cytometry

PBMCs were isolated from participants blood samples and frozen at the 1b trial sites in Burkina Faso and Madagascar. Upon receipt at Texas Tech University Health Sciences Center (TTUHSC), the cells were stored in liquid nitrogen vapor phase. The immunization scheme adopted for all three trials is illustrated in Fig. 1A. Frozen PBMCs were thawed and washed, then assessed for cell count and viability. Viability was between 80% and 95%. The cells were suspended in complete RPMI-1640 medium with ATCC modification (Gibco A10491-01) (10% FBS Gibco + antibiotic antimycotic cocktail sigma, A5955) and allowed to acclimatize for 2 hours. Following a 2 hour incubation, cells were washed with an excess of complete culture medium by centrifugation at 300×*g* for 5 minutes and seeded into 48-well culture plates at density of 2×10^6^ cells/mL.

PBMCs collected from all eight time points (Fig. 1A) were cultured in vitro simultaneously. Each PBMC sample was split into two sets: one set was left untreated, while the second set was stimulated with 12 µg of Sm-p80 antigen to examine recall responses. PBMCs were cultured for 72 hours. Subsequently, cells were analyzed by flow cytometry while supernatants were collected to determine secreted cytokine levels (Supplementary Fig. 1). Various Sm-p80 antigen concentrations and culture durations (24, 48, and 72 hours) were tested (not shown). A 72-hour culture duration and 12 µg of Sm-p80 antigen performed optimally for measuring recall responses in vitro. Since the PBMC were shipped frozen from various trial sites, prior to performing actual experiments, we tested the reactivation of PBMCs using the 72-hour culture condition with Dynabeads™ Human T-Activator CD3/CD28 for T Cell Expansion and Activation (ThermoFisher). The CD4^+^ and CD8^+^ T-cells were able to become activated normally as demonstrated by CD69 expression (Supplementary Fig. 2A, B).

Staining and flow cytometry analysis. On day 3 of culture, cells were removed from the incubator and transferred to a clean 5 mL polystyrene tube. The culture supernatants were collected in clean 1.6 mL MCT and stored at -80˚C for cytokine analysis. For antibody staining, the cells were initially washed with 1 mL FACS buffer (1× PBS plus 0.5% BSA) by centrifuging at 300×*g* for 5 minutes. Following which, cell pellets were resuspended in 1× solution of BD Horizon™ Brilliant Stain Buffer (Cat# 563794) containing FcR blocking antibody (Cat. #422302, Biolegend, Human TruStain FcX™), supplemented with an antibody cocktail for either effector and memory T- or B- plasma cell panels. Cells were stained with the respective antibody cocktails for 45-minuites on ice. Following incubation, cells were washed with FACS buffer and the resulting pellet was suspended in 100 µl of PBS prior to data acquisition on a full spectrum flow cytometer (Northern lights by Cytek USA, 3 Lasers). A minimum of 100,000 P1 events were acquired. Data analysis was performed using SpectroFlo^®^ v3.01 (Cytek USA). The frequency of Sm-p80 antigen specific CD4^+^ effector memory population was estimated in naïve and non-naïve endemic samples by subtracting the background effector memory population present at baseline (day 0, Sm-p80 stimulated)^62,63^.

### ELISpot

ELISpot assays were performed using the Immunospot HuIgG kit (Cat. # hIgG-SCE-2M/2, Cellular Technologies Limited). We applied a modified protocol per manufacturer recommendations to detect Sm-p80 specific antibody secreting cells/plasmablasts. Briefly, PBMCs from human volunteers were plated at density of 2×10^6^ cells/mL overnight in assay medium as recommended. ELISpot plate activation involved a pre-wetting step followed by antigen coating. The plate was coated using 80 µl of Sm-p80 antigen solution at 10 µg/mL, sealed, and incubated overnight at 4 °C. Next day, plate was washed and blocked with 1% Bovine Serum Albumin (BSA) in 1×PBS. After one hour blocking step, plate was washed, and 100 µL of assay media containing cells were added to the plate and incubated for 4-8 hours. After incubation, the plate was washed and subjected to the detection protocol. Finally, the plate was dried overnight in a running laminar flow hood and scanned on ELISpot Bioreader 5000 (BIO-SYS GmbH).

### Statistical analysis

For in vitro data analysis requiring statistical analysis, we applied averages ± SEM. Paired Wilcoxon signed rank test was applied for pre-vaccinated and post-vaccination intra group comparisons. A paired Student’s t-test was used for pairwise comparisons and one way ANOVA was applied for longitudinal data. P values less than 0.05 were considered statistically significant. Statistical analyses were performed using GraphPad Prism and SPSS (IBM).

## Supplementary information


Supplementary information 4 9 26.


## Data Availability

The datasets generated and/or analyzed during the current study are not currently publicly available due to the human subject confidentiality, to protect any identifiable research data and to prevent unauthorized access or disclosure. However, datasets are available from the first author (MYB) and the senior author (AAS) upon reasonable request, including individual de-identified participant data (including data dictionary) and statistical code.
