# Supplementary material for: Schistosomiasis vaccine SchistoShield® induces functional immune memory responses in US and African populations
Source: NPJ Vaccines. 2026 Jun 2;11:173. doi: 10.1038/s41541-026-01501-0 (PMC13526879; doi:10.1038/s41541-026-01501-0)
Supplement: Supplementary file 1 [file 41541_2026_1501_MOESM1_ESM.pdf]

Supplemental Information

Schistosomiasis vaccine SchistoShield® induces functional immune memory responses in US and African populations

Mumtaz Y. Balkhi[1,2], Aravindan Kalyanasundaram[1,2†], Aryandra Arya[1,2†], Desalegn W. Kifle[1,2], Mallory Mckinnon[1,2], Dinesh Krishnappa[3], Harshvardhan Shrivastava[3], Mohamadou Siribie[3], Asma Aziz[3], Hyonjin Jeon[3,4,5], Tanyada Yongchaitrakul[3], Birkneh T. Tadesse[3,6], Njariharinjakamampionona Rakotozandrindrainy[4], Ravomialisoa Razafimanantsoa[4], Henry Bere Noellie[7], Nebié Issa Ouedraogo[7], Amidou Diarra[7], Sean A. Gray[8], Rhea N. Coler[9], Jinhee Lee[10], Raphaël Rakotozandrindrainy[4], Sodiomon B. Sirima[7], Lisa A. Jackson[11], Florian Marks[3,4,5,6,12], Darrick Carter[8] and Afzal A. Siddiqui[1,2*]

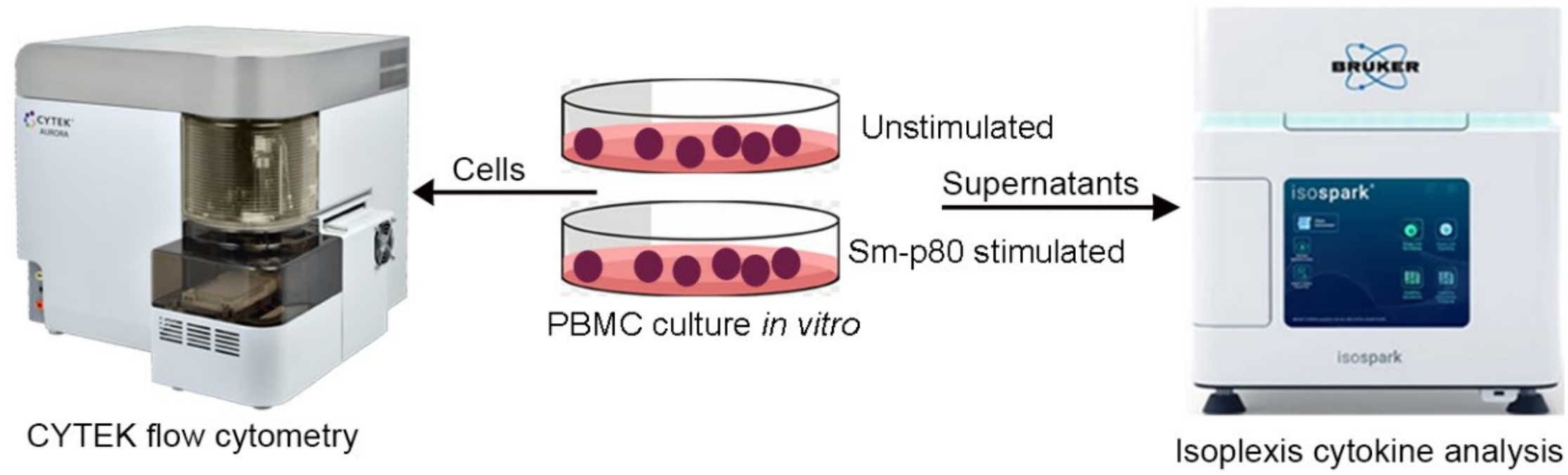

Supplementary Figure 1. Illustrates the methodology adopted to study immune memory and effector cytokine response. Across all three trials, PBMCs were cultured in presence or absence of Sm-p80. The supernatants were collected and analyzed for cytokine production using IsoPlexis chips. Cells were stained with fluorochrome conjugated antibodies to determine T and B cell profile using Cytek flow cytometer.

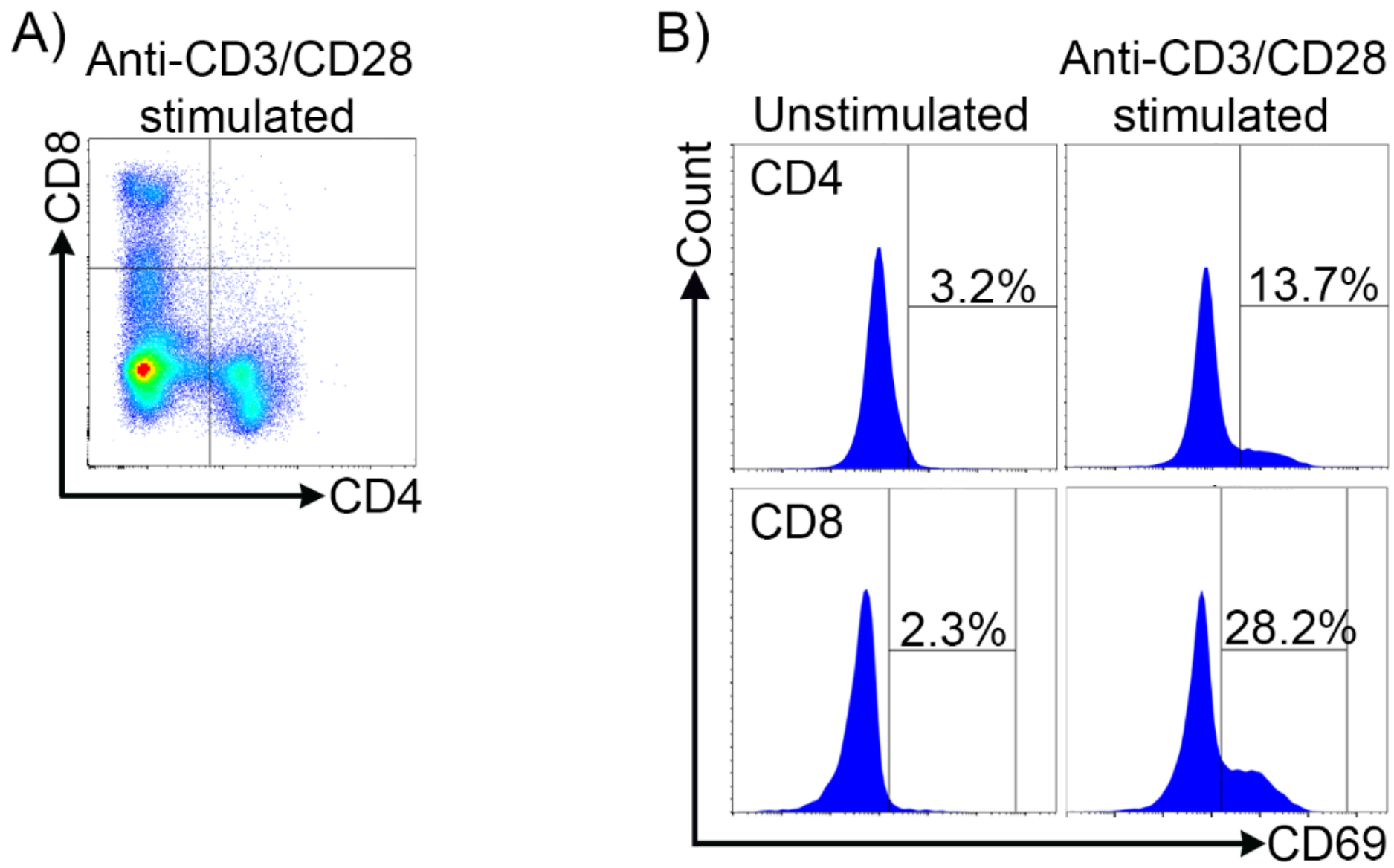

Supplementary Figure 2. PBMCs shipped frozen from African trial sites were analyzed through flow cytometry to determine their reactivation potential with human T cell activator CD3/CD28 dyna- beads. PBMCs were cultured in presence or absence of dynabeads maintaining 1:1 (cell to bead ratio). After 3-days in culture, beads were removed using a magnet and cells stained with CD4, CD8 and CD69. **(A-B)** $CD4^+$ and $CD8^+$ T cells were then analyzed for CD69 upregulation.

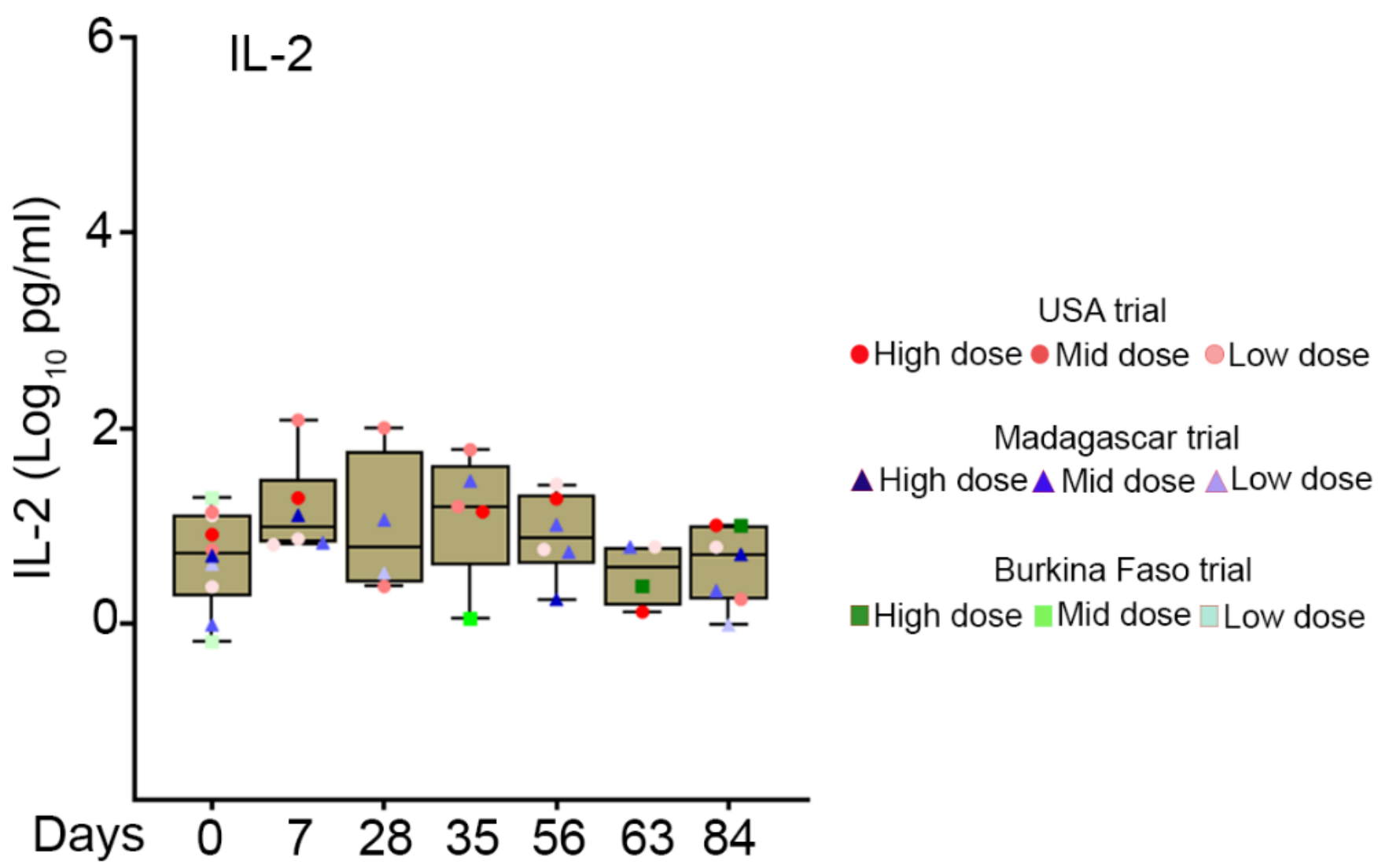

Supplementary Figure 3. SchistoShield® vaccine induced IL-2 cytokine production. PBMC culture supernatants (unstimulated) were analyzed for IL-2 production using Isoplexis chips. As shown, post primary vaccination (day 7), and one week post first booster dose (day 35), the production of IL-2 cytokine increased relative day 0.

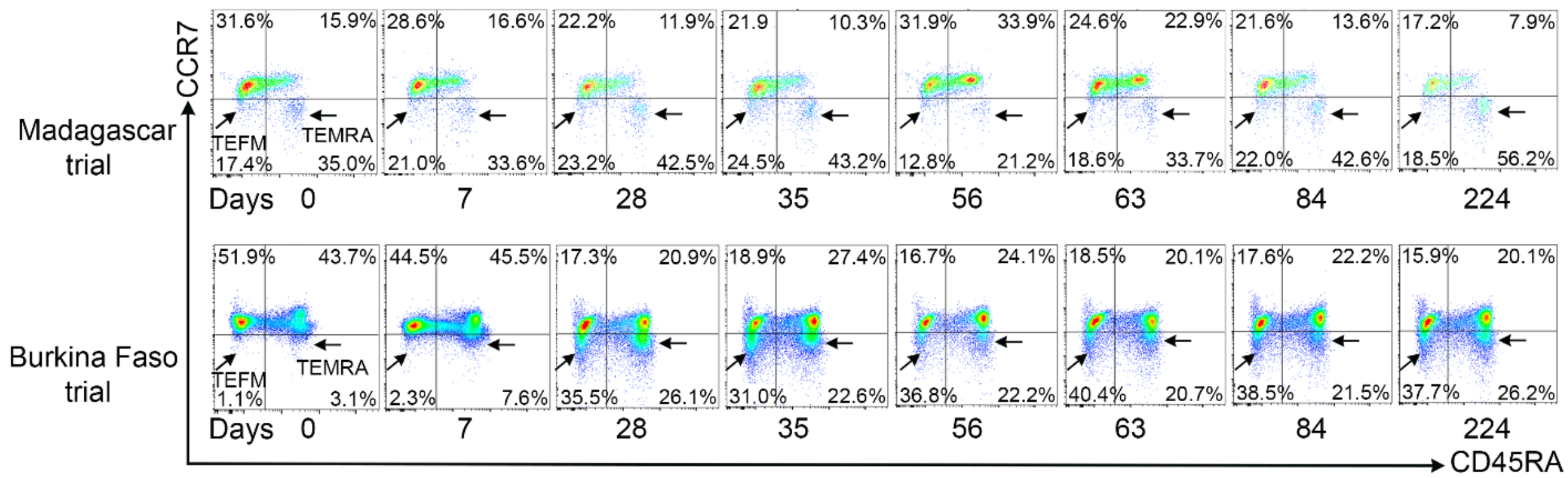

Supplementary Figure 4. SchistoShield® vaccine expands CD8+ TEMRA and effector memory cells. A representative flow cytometry profile of Madagascar and Burkina Faso CD8+ memory cells in volunteers vaccinated with mid dose SchistoShield® vaccine. The profile obtained is similar to volunteers vaccinated with low or high dose SchistoShield®.

Days	0	7	28	35	56	63	84	180/224
IFN-γ	303.9	940.3	604.1	454.2	760.0	593.9	676.3	323.8
TNF-α	97.9	283.7	339.8	211.3	718.9	856.0	678.1	132.9
IL-9	74.7	108.5	105.2	98.5	74.0	102.9	90.3	44.2
IL-17A	5.0	21.2	28.9	22.8	3.6	5.3	6.3	2.7
Granzyme B	474.2	512.1	744.5	983.9	529.9	448.1	751.9	535.7

Supplementary Table 1. Cytokine levels (pg/mL) detected in the volunteer PBMC culture supernatants following Sm-p80 stimulation. Values represent the combined mean cytokine concentrations derived from USA, Madagascar and Burkina Faso clinical trial samples. Cytokines were detected using CodePlex/IsoPlexis chips and analyzed via IsoSpeak software.
